# Animal Models of Chronic Cerebral Hypoperfusion: From Mouse to Primate

**DOI:** 10.3390/ijms20246176

**Published:** 2019-12-07

**Authors:** Kazuo Washida, Yorito Hattori, Masafumi Ihara

**Affiliations:** Department of Neurology, National Cerebral and Cardiovascular Center, Suita 564-8565, Japan; yoh2019@ncvc.go.jp (Y.H.); ihara@ncvc.go.jp (M.I.)

**Keywords:** vascular cognitive impairment, chronic cerebral hypoperfusion, rat model of bilateral carotid artery occlusion, mouse model of bilateral common carotid artery stenosis, mouse model of asymmetric carotid artery surgery, non-human primate model of 3-vessel occlusion

## Abstract

Vascular cognitive impairment (VCI) or vascular dementia occurs as a result of brain ischemia and represents the second most common type of dementia after Alzheimer’s disease. To explore the underlying mechanisms of VCI, several animal models of chronic cerebral hypoperfusion have been developed in rats, mice, and primates. We established a mouse model of chronic cerebral hypoperfusion by narrowing the bilateral common carotid arteries with microcoils, eventually resulting in hippocampal atrophy. In addition, a mouse model of white matter infarct-related damage with cognitive and motor dysfunction has also been established by asymmetric common carotid artery surgery. Although most experiments studying chronic cerebral hypoperfusion have been performed in rodents because of the ease of handling and greater ethical acceptability, non-human primates appear to represent the best model for the study of VCI, due to their similarities in much larger white matter volume and amyloid β depositions like humans. Therefore, we also recently developed a baboon model of VCI through three-vessel occlusion (both the internal carotid arteries and the left vertebral artery). In this review, several animal models of chronic cerebral hypoperfusion, from mouse to primate, are extensively discussed to aid in better understanding of pathophysiology of VCI.

## 1. Introduction

Vascular cognitive impairment (VCI) or vascular dementia occurs as a result of persistently compromised blood flow to the brain and represents the second leading cause of dementia after Alzheimer’s disease [1,2]. However, the precise pathophysiological mechanisms underlying VCI remain unclear despite extensive research [3,4].

VCI results from systemic, cardiac, or local large or small vessel disease [5,6]. There are several causes of VCI: cerebral small vessel disease, multi-infarct dementia; strategic infarct (i.e., located in a functionally-critical brain area), hemorrhage/microbleed; angiopathy (including cerebral amyloid angiopathy), severe hypoperfusion (e.g., heart failure, cardiac arrhythmia), and hereditary vasculopathy (e.g., cerebral autosomal dominant arteriopathy with subcortical infarcts and leukoencephalopathy, CADASIL) [7,8]. No single animal model can replicate them all [9]. For instance, animal models of chronic cerebral hypoperfusion show demyelination and axonal damage, but do not show cardiovascular or other neurobiological changes of VCI, such as heart failure, hypertension, or arterial pathologies such as arteriosclerosis, fibrinoid necrosis, and lipohyalinosis [10]. However, in this review, we exclusively focused on chronic cerebral hypoperfusion animal models since subcortical vascular dementia, primarily characterized by ischemic white matter lesions due to chronic cerebral hypoperfusion, accounts for approximately half of all VCI cases in humans [10].

The cerebral blood flow (CBF) is diffusely or at least focally reduced in elderly patients with VCI [11]. Accordingly, for investigation of the mechanisms underlying VCI, several models of chronic cerebral hypoperfusion have been established in rats [12], mice [13,14], and non-human primates [15]. Models of chronic cerebral hypoperfusion can be generated by bilateral common carotid artery (CCA) occlusion in rats, bilateral CCA stenosis in mice, asymmetric CCA surgery in mice, and bilateral internal carotid artery and left vertebral artery occlusion (three-vessel occlusion; 3VO) in non-human primates. These animal models also provide unique platforms for the investigation of potential drugs or cell therapies, considering that no disease-modifying drugs for VCI have been developed till date [16].

In this review, several animal models of chronic cerebral hypoperfusion, ranging from mice to primates, are extensively discussed to further the understanding of pathophysiological mechanisms underlying VCI. Specifically, we have discussed the characteristic details accumulated since the establishment of rat, mice, and primate models of chronic cerebral hypoperfusion, with a focus on the methods, CBF, histopathological changes, cognition, motor functions, and practical applications.

## 2. Pathogenetic and Neurobiological Mechanism of Subcortical Vascular Cognitive Impairment

**Figure 1:**
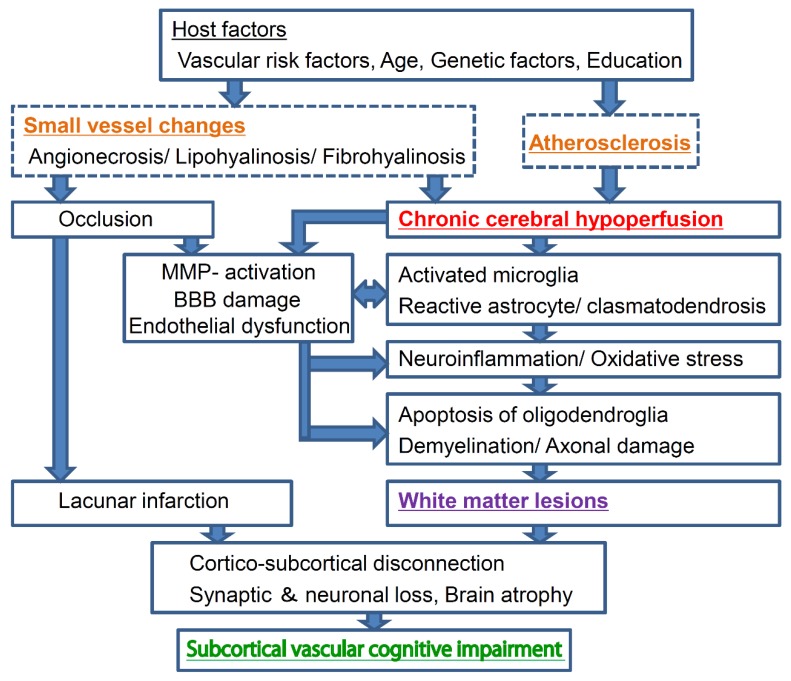
Schema of the pathogenetic and neurobiological mechanism of subcortical vascular cognitive impairment. Subcortical vascular cognitive impairment is mainly caused by chronic cerebral hypoperfusion and small vessel disease. Vascular risk factors, such as hypertension, diabetes mellitus, and aging, cause small vessel changes and atherosclerosis. Small vessel change may cause its occlusion and lacunar infarction, whereas loss of vasomotor reactivity due to angionecrosis/lipohyalinosis/fibrohyalinosis in the small vessels may cause chronic cerebral hypoperfusion in the white matter. Atherosclerosis also causes chronic cerebral hypoperfusion. Subsequent microglial/astroglial activation, matrix metalloproteinase activation, blood-brain barrier damage, and endothelial dysfunction are likely to induce neuroinflammation, oxidative stress, oligodendroglial apoptosis, and thence white matter lesions.

Subcortical VCI accounts for approximately half of all VCI cases in humans [10]. The pathogenetic and neurobiological mechanism of subcortical VCI is illustrated for a deeper understanding (Figure 1). Subcortical VCI is mainly caused by chronic cerebral hypoperfusion and small vessel disease. Vascular risk factors, such as hypertension, diabetes mellitus, and aging, cause small vessel changes, and atherosclerosis. Small vessel change may cause its occlusion and lacunar infarction, whereas loss of vasomotor reactivity due to angionecrosis/lipohyalinosis/fibrohyalinosis in the small vessels may cause chronic cerebral hypoperfusion in the white matter. Atherosclerosis also causes chronic cerebral hypoperfusion. Subsequent microglial/astroglial activation, matrix metalloproteinase activation, blood-brain barrier damage, and endothelial dysfunction are likely to induce neuroinflammation, oxidative stress, and oligoendroglial apoptosis, leading to white matter lesions [17,18,19,20].

Animal models of chronic cerebral hypoperfusion are used as subcortical VCI models and do not fully explain all aspects of VCI in humans. VCI also results from cardiovascular mechanisms such as heart failure, hypertension, cardiac arrythmia, arterial sclerosis, cerebral amyloid angiopathy, transient ischemia, focal ischemia, embolism, and hereditary vasculopathy (e.g., CADASIL) [8,17,18]. For instance, animal models of chronic cerebral hypoperfusion show white matter lesions and cognitive impairment, but do not correspond well to the cardiovascular mechanisms of VCI. To make up for this drawback, animal models of cardiovascular diseases such as heart failure (transverse aortic constriction model [21,22,23,24], myocardial infarction model [25]), hypertension (stroke prone spontaneously hypertensive rat model [26,27], chronically hypertensive rhesus monkey model [28]), atherosclerosis (Apolipoprotein E knockout mouse model [29,30], low-density lipoprotein receptor-knockout mouse model [31]), and embolic models [32,33] are essential for a deeper understanding of vascular dementia in humans [17,18].

## 3. Rat Model of Chronic Cerebral Hypoperfusion

Four animal models of chronic cerebral hypoperfusion are outlined in this review (Table 1). Difficulty, time, and cost of surgery of each animal model are also outlined (Table 2). First, we will discuss the rat model, which can be generated by bilateral CCA occlusion (BCAO). The rat model of chronic cerebral hypoperfusion has been used for several years and is one of the most widely used and established experimental model [7,34], exhibiting cognitive impairment and cholinergic deficits in addition to white matter rarefaction resembling those associated with cerebral white matter lesions induced by chronic cerebral hypoperfusion in humans. In fact, cerebral hypoperfusion, induced by carotid artery stenosis [35,36], atrial fibrillation [37,38], and heart failure [39], is associated with cognitive decline in the general population [40]. Interestingly, BCAO in the rat could also be used as a model of retinal ischemia [41]. Although the optic nerve damage is strong, it has been used as a hypoperfusive subcortical VCI model. However, this model also has some inherent limitations. For example, the visual pathway is invariably damaged by occlusion of the ophthalmic arteries [41], and this potentially compromises behavioral assessments. Furthermore, genetic studies may be hampered because of limited accessibility to molecular technologies for rats.

### 3.1. Method

Rat models of chronic cerebral hypoperfusion are prepared using male Wistar rats aged 9–12 weeks (weight, 300–350 g). Under anesthesia with 1–3% halothane inhalation, both CCAs are exposed through a midline cervical incision and permanently ligated [42]. Only CCAs should be ligated after careful incision of the carotid artery sheath followed by separation of the vagus nerve from CCA. Although the survival rate is generally ≥90%, stimulation of the vagus nerve during surgery should be avoided as far as possible. Moreover, the body temperature should be maintained using a heat retention pad.

This surgery is relatively easy since we just need to ligate both CCAs with sutures and it only takes 10 min to perform (Table 2). The cost is only incurred from the sutures.

### 3.2. Cerebral Blood Flow

CBF is strongly reduced in the acute ischemic phase after surgery. This is one of the limitations of rat models. CBF in the cortex and corpus callosum was shown to reduce to 30–50% of the original level at 1 to 3 days after surgery, with gradual recovery after 3–8 days following surgery [43]. In the chronic ischemic phase, CBF values slowly normalize by 1 week, although they remain significantly lower than the control values at 4 weeks after surgery. By 8 weeks to 3 months after surgery, CBF is almost restored to the original level. The blood flow reduction in the hippocampus and thalamus is slightly lesser than that in the cerebral cortex, reducing to 50–70% and recovering to the original level by 3 months [34].

### 3.3. White Matter Lesions

The oligodendrocyte exhibits apoptosis and white matter rarefaction after 14 days. In the white matter, microglia are increased and activated from the first postoperative day. Reactivity changes in the astroglia are also observed after the third postoperative day. These activated glial cells produce inflammatory cytokines and free radicals and may play a role in white matter disorders [43]. White matter lesions primarily based on demyelination develop in the optic tract after 3 days and corpus callosum after 7 days.

### 3.4. Cortical Lesions

Cortical gray matter disorders vary with age, lineage, and surgical technique, and there is a discrepancy among reports [34]. Although no morphological abnormalities have been found in the cerebral cortex and hippocampus, one study reported mild cell shedding mainly in the hippocampal CA1 region [44].

### 3.5. Behavioral Analysis

In the BCAO rat model, because the optic nerve damage is strong, attention should be paid to interpretation of the results of behavioral assessments dependent on spatial working memory. Spatial learning ability assessed by the Morris water maze test was found to be impaired at 4 weeks after surgery, with more severe impairment at 20 weeks [45], while spatial working memory impairment was evident in the eight-arm radial maze test at 16 months. With regard to the object recognition test, abnormalities that were not observed at 30 days became evident at 60 and 90 days after surgery. Therefore, it is speculated that cognitive dysfunction cannot be attributed to acute-phase ischemic changes, whereas the influence of the chronic phase seems to be great.

### 3.6. Sensory/Motor Function

No major sensory/motor deficit has been reported in rat models [7].

## 4. Mouse Model of Chronic Cerebral Hypoperfusion (Bilateral Common Carotid Artery Stenosis)

As described above, the rat model of chronic cerebral hypoperfusion possesses some inherent drawbacks such as optic nerve damage and difficulty in genetic assessments. To overcome these limitations, more appropriate animal models are desirable. Accordingly, a mouse model of chronic cerebral hypoperfusion induced by bilateral CCA stenosis (BCAS) was established [13,46,47]. This model does not involve optic nerve damage and is therefore suitable for behavioral experiments; moreover, genetically modified animals can be used. In recent years, it has been increasingly used as a stable subcortical VCI model [18]. The mortality rate of the BCAS model, when performed by trained researchers, is less than 2%. Now that the BCAS model is used worldwide, it can be regarded as one of the most promising models of chronic cerebral hypoperfusion [18].

The severity of cerebral hypoperfusion can be easily controlled by regulation of the internal diameter of microcoils. This model mimics a neurological clinical condition of white matter rarefaction associated with white matter lesions induced by chronic cerebral hypoperfusion in humans. In fact, cerebral hypoperfusion, induced by carotid artery stenosis [35,36], atrial fibrillation [37,38], and heart failure [39], is associated with cognitive decline in the general population [40]. The model develops white matter lesions without cerebral infarctions and optic nerve damage, while hippocampal atrophy is induced for a longer time period. Interestingly, this may accurately reflect the hippocampal atrophy that is often observed in human patients with pure hypoperfusive subcortical VCI [48].

The apparent advantage of using this surgical technique is that it provides a unique platform to investigate the precise pathophysiological mechanism underlying VCI and explore potential drugs or cell therapies designed to develop novel treatments for dementia of vascular origin.

However, the mouse model of BCAS-induced chronic cerebral hypoperfusion has several limitations, including the lack of motor dysfunction and abrupt CBF reduction after the surgery [46,49].

To overcome these limitations, a better mouse model with motor impairment and a gradual reduction in CBF was recently established [14]; this will be discussed later. Nevertheless, a non-human primate model is always desirable because the rodent brain is different from the human brain in that it exhibits an extremely small volume of white matter [50] and no amyloid β accumulation.

### 4.1. Method

The mouse model of chronic cerebral hypoperfusion is developed in male C57BL/6J mice aged 10–12 weeks (weight, 24–29 g). We did not apply BCAS surgery to Wistar rat for several reasons. First, we don’t have appropriately sized microcoils for rat surgery. Second, the rat model of chronic cerebral hypoperfusion has some inherent drawbacks such as optic nerve damage and difficulty in genetic assessments. Third, the Wistar rat is more resistant to cerebral ischemia than the C57Bl/6J mouse [51]. Among seven mouse strains, we selected the C57Bl/6J mouse strain as it is most vulnerable to cerebral ischemia [13]. It has been hypothesized that the gradual recovery of cerebral blood flow is aided by blood supply through the posterior communicating artery. Therefore, a poorly-developed posterior communicating artery is suitable for maintaining chronic cerebral hypoperfusion over an extended period [14]. All procedures in this surgery were carried out in strict accordance with the guidelines for animal experimentation from the Animal Research Committee of Kyoto University. The protocol was approved by the Animal Research Committee, Kyoto University (Permit Number: MedKyo13270). All surgery was performed under anesthesia, and all efforts were made to minimize suffering.

**Figure 2:**
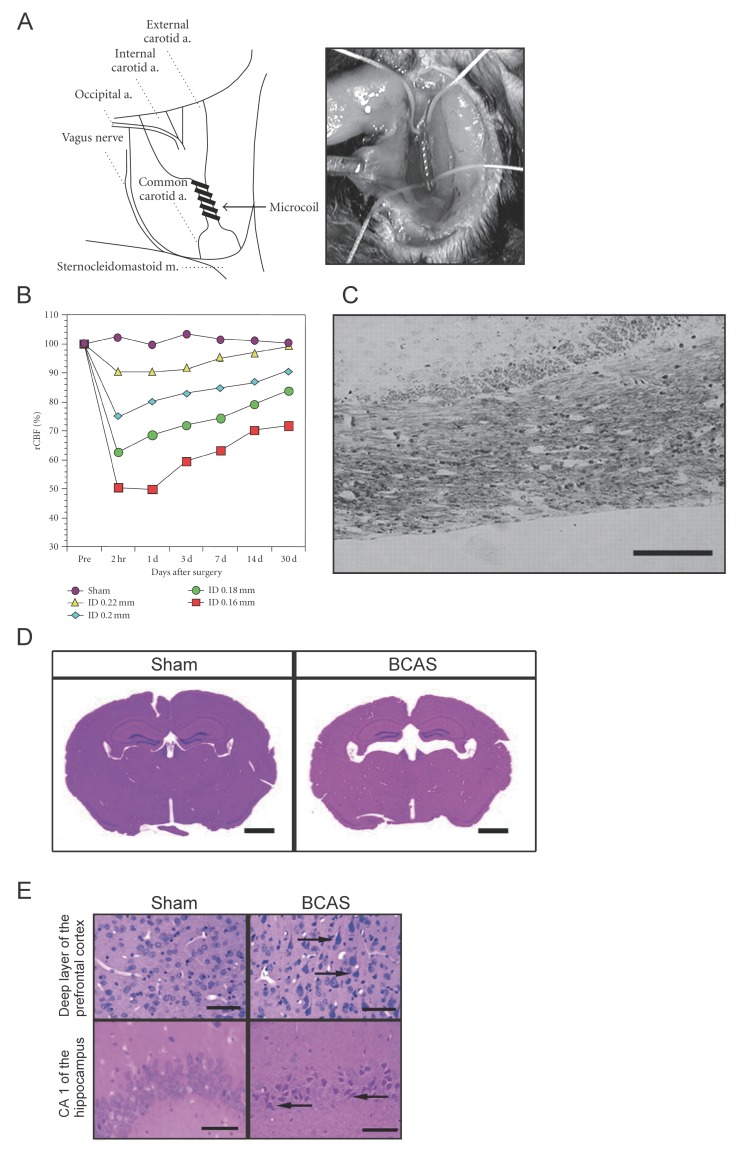
Mouse model of chronic cerebral hypoperfusion induced by bilateral common carotid artery (CCA) stenosis (BCAS). (**A**) The procedure for BCAS and microcoil placement in a C57BL/6J mouse. The microcoil is twined by rotation around CCA just proximal to the carotid bifurcation. (**B**) Cerebral blood flow (CBF) values after BCAS. The data are expressed as means ± standard deviations (SDs) or percentages of the preoperative values. * *p* < 0.05, ** *p* < 0.01 compared with control values. (**C**) There is myelin rarefaction and vacuole formation in the corpus callosum after placement of a microcoil with an inner diameter of 0.18 mm. Scale bar, 100 μm. (**D**) Representative hematoxylin and eosin-stained sections obtained from the sham (left) and BCAS (right) groups. Note the resultant dilation of the ventricle in the BCAS group. Scale bars, 1 mm. (**E**) Representative images of the deep layer of the frontal cerebral cortex (upper panels) and the hippocampal CA1 region (lower panels) for the sham (left panels) and BCAS (right panels) groups. The arrows indicate pyknotic neurons. Scale bar, 50 μm [13,52].

Under anesthesia with 2% halothane inhalation, both CCAs are exposed through a midline cervical incision, followed by the application of a microcoil with an inner diameter of 0.18 mm (Figure 2A) [13]. Similar to the procedure used for the rat model of BCAO, the carotid artery sheath is dissected and the vagus nerve is separated from CCA so that the microcoil is applied only to CCA. The microcoil consists of piano wires with an internal diameter of 0.18 mm and is manufactured by mounting a microcoil (pitch, 0.5 mm; total length, 2.5 mm). Several microcoils with inner diameters varying from 0.16 mm to 0.22 mm are available for use. The survival rate at 1 week after surgery using a microcoil with a 0.18-mm inner diameter is ≥85%. The rectal temperature should be maintained between 36.5 °C and 37.5 °C during the procedure.

The difficulty of this surgery is moderate: microcoils have to be placed on both CCAs and it takes 10 min to perform. Microcoils can be purchased from Wuxi Samini Co., Ltd. (Wuxi, China). The cost of one is $25. Therefore, it costs $50 (two microcoils) per surgery (Table 2).

### 4.2. Cerebral Blood Flow

The mouse model also exhibits a strong reduction in CBF in the acute phase of ischemia after surgery. However, CBF in this model can be manipulated by changing the inner diameter of the microcoil. A microcoil with a 0.18-mm diameter, which is generally used from the perspective of reproducibility and confidence, results in a 30% drop in CBF at 2 h after surgery, following which there is gradual recovery (Figure 2B) [13]. A study showed that the blood flow in the cerebral cortex temporarily decreased to as much as 60% to 70% of the control value at 2 h after surgery, with gradual recovery to >80% at 1–3 months [52].

A major limitation of the mouse model is a relatively strong decrease in CBF in the acute phase after surgery. In other words, it remains unclear whether actual chronic cerebral hypoperfusion, characterized by both acute- and chronic-phase tissue changes, develops at 1–3 days after surgery. Accordingly, a model that does not exhibit such a strong reduction in CBF would be preferable.

### 4.3. White Matter Lesions

There is strong white matter rarefaction in the following order: corpus callosum > striatum > internal capsule > optic tract. This change appears in the corpus callosum, striatum, and internal capsule at 14 days after BCAS. Moreover, activation of the microglia and astrocyte proliferation are observed after 7 and 14 days following BCAS, respectively (Figure 2C) [13]. At the same time, the microglia exhibit a significant increase from 7 to 30 days after BCAS, whereas the astroglia increase from 14 to 30 days. Edema also appears to play a role in the development of white matter lesions. Activation of matrix metalloproteinase (MMP), which induces decomposition of the constituent proteins of the vascular basement membrane, impairment of the blood–brain barrier (BBB), and extravasation of serum proteins is also observed [53]. Among MMPs, MMP-2 has a strong ability to degrade myelin basic protein, and it is also believed to cause direct damage to the white matter.

### 4.4. Cortical Lesions

Gray matter lesions do not appear after BCAS using microcoils with an inner diameter of ≥0.18-mm in mice. However, microinfarctions in the cerebral cortex, cell shedding centered on the hippocampal CA1 region, and necrotic lesions scattered in the basal ganglia appear after most BCAS procedures using microcoils with an inner diameter of 0.16 mm, with a significant increase in the mortality rate [13].

The mouse model demonstrates good reproducibility in terms of glial activation, BBB disruption, and the development of white matter lesions and cognitive dysfunction, which appear within a month after surgery. Furthermore, in the longer term, significant hippocampal atrophy with pyknotic and apoptotic cells are observed at 8 months after surgery; this is evidence of a link between chronic cerebral hypoperfusion and neurodegeneration (Figure 2D,E) [52]. Hippocampal atrophy is induced by damage to the white matter that intimately connects with the hippocampus, and this damage can indirectly induce hippocampal atrophy through remote effects [52]. Actually, CBF in the hippocampus is significantly reduced after BCAS [54]. Interestingly, this may accurately reflect the hippocampal atrophy that is often observed in human patients with subcortical vascular dementia [48]. These results suggest that, in the long term, the mouse model of BCAS-induced chronic cerebral hypoperfusion replicates advanced stages of subcortical ischemic vascular dementia characterized by significant hippocampal neuronal loss.

### 4.5. Behavioral Analysis

Unlike the BCAO rat model, the BCAS mouse model shows constant blood flow in the common cervical and internal carotid arteries; therefore, the blood flow in the branched ocular artery is also preserved, resulting in preservation of the optic nerve. The white matter, which is the main component of the frontal-subcortical brain circuits, is strongly associated with the processing speed. Spatial working memory impairment is a characteristic feature of this model, because working memory depends on white matter integrity [52]. A decrease in spatial working memory is observed in the eight-arm radial maze test performed at 1 month after surgery, although reference memory impairment is not observed. This reproduces the cognitive profile of VCI in humans and is considered cognitive dysfunction due to impaired fiber communication between the frontal lobe and basal ganglia. However, in the longer term, i.e., at 5–6 months after BCAS, both reference and working memory are impaired in the Barnes and radial arm maze tests, respectively [52]; this is induced by both white matter lesions and hippocampal changes.

### 4.6. Sensory/Motor Function

No major sensory/motor deficit can be observed before three months after BCAS [46]. However, despite the absence of lacunar infarcts, mice were found to exhibit impaired motor function at three months after BCAS, thus reproducing one of the key features of subcortical vascular dementia. It was speculated that frontal-subcortical circuit disruption may have resulted in not only cognitive impairment but also motor dysfunction [52].

### 4.7. Pharmacological Experiments and Applications to Genetically Modified Animals

Till date, the development of various medicines and therapies has involved the use of the BCAS mouse model. These include angiotensin II type 1 receptor [55], phosphodiesterase III inhibitor [56], bone marrow mononuclear cells [57], and environmental enrichment therapy [58,59], which reportedly protect the white matter. Their clinical application is yet awaited.

Furthermore, the BCAS procedure is easy to apply to genetically modified mice, and there have been some applications thus far. Cerebral hypoperfusion is also a common underlying mechanism and a major contributor to cognitive decline and degenerative processes in Alzheimer’s disease [60,61,62]. When chronic cerebral hypoperfusion was induced in mouse models of Alzheimer’s disease, represented by mice overexpressing a mutant form of the human amyloid precursor protein bearing both the Swedish and Indiana mutations (APP (Sw/Ind)-Tg mice), it was found that the coagulability of the amyloid protein was enhanced. These results suggest an interaction between chronic cerebral hypoperfusion and APP (Sw/Ind) overexpression in the cognitive decline caused by enhanced neuronal loss and altered amyloid β metabolism in mice [63]. Thus, the ease of application of BCAS in genetically modified animals is expected to clarify the pathology of ischemic white matter lesions and aid in the development of therapies for neurodegenerative diseases such as Alzheimer’s disease. To summarize, the mouse model of BCAS-induced chronic cerebral hypoperfusion is currently considered the best animal model of VCI because of its reproducibility and genetic applicability [18,64,65].

In the prospect of initiating translational research, we will begin an investigator-initiated clinical trial based on the evidence obtained from the BCAS mouse model. Adrenomedulin (AM), a vasoactive peptide hormone, is secreted from endothelial cells in various organs such as the brain, adrenal medulla, heart, and kidney [66]. AM has a variety of effects on vasculature including vasodilation, permeability regulation, inhibition of endothelial cell apoptosis and oxidative stress, smooth muscle cell proliferation regulation, and angiogenesis promotion [66]. By subjecting mice to overexpress circulating AM in BCAS, we previously assessed its effects of AM on cerebral perfusion, cerebral angioarchitecture, oxidative stress, white matter change, and cognitive function [67]. In addition, an experimental study using a mouse model of middle cerebral artery occlusion demonstrated the protective effects of AM against ischemic stroke by promoting angiogenesis and suppressing cerebral edema [68,69]. Based on these results, we will start an investigator-led clinical trial for patients with ischemic stroke to confirm the effects of AM on acute cerebral ischemia.

## 5. Mouse Model of Chronic Cerebral Hypoperfusion (Asymmetric Common Carotid Artery Surgery)

In the mouse model of chronic cerebral hypoperfusion induced by BCAS, the subcortical white matter is a frequent target of ischemic injury. Extensive white matter lesions are important substrates of VCI in humans. However, other procedures for generating rodent models of ischemic stroke mainly induce cerebral infarcts in the gray matter, while BCAS in mice only results in white matter rarefaction without infarcts [52]. The lack of animal models that consistently replicate white matter infarct-related damage may partially explain why many neuroprotective drugs for VCI have shown clinical failure despite success in preclinical experiments [16].

The other limitation of the BCAS mouse model is that the decline in CBF is relatively abrupt, and this does not replicate the normal pattern of VCI in humans. Furthermore, this model only exhibits cognitive impairment without motor dysfunction in the first 3 months after surgery. Thus, it does not describe all the features of subcortical vascular dementia [17].

Accordingly, a mouse model of white matter infarct-related damage with cognitive and motor dysfunction induced by asymmetric CCA surgery (ACAS) was established [14]. This model can be generated by surgical implantation of different devices in the right and left CCAs in C57BL/6J mice. Implantation of an ameroid constrictor in the right CCA results in gradual occlusion of the vessel over 28 days, whereas placement of a microcoil in the left CCA induces approximately 50% arterial stenosis. Moreover, gradual CBF reduction is observed in this model, with the induction of white matter infarcts, rarefaction, and gliosis. Furthermore, motor impairments, including gait disturbance and muscle weakness, develop before executive dysfunction mimicking a neurological clinical condition of chronic cerebral hypoperfusion in humans (e.g., carotid artery stenosis [35,36], atrial fibrillation [37,38], and heart failure [39]). The existing rodent models of chronic cerebral hypoperfusion partially recapitulate subcortical VCI by developing white matter rarefaction [17]. However, the BCAO rat model and BCAS mouse model do not show other characteristics of subcortical VCI, such as infarctions as well as gradual CBF reduction. Another advantage of the ACAS model would be right-to-left comparison of efficacy of certain drugs on ischemia because of the difference in ischemic depth between the hemispheres. The right hemisphere mimics the characteristics of multiple subcortical infarcts and the left hemisphere mimics leukoaraiosis, sequelae to chronic cerebral hypoperfusion. Prompt and reproducible generation of small subcortical infarcts and leukoaraiosis, in a timely manner would be an advantage in the analysis of subcortical VCI pathophysiology and search for drugs that target subcortical VCI. Thus, the ACAS model represents a novel animal model, which accurately replicates predominant white matter infarcts accompanied by motor deficits and dementia, similar to hypoperfusive subcortical VCI with infarctions and motor deficits, like Binswanger’s disease [14].

### 5.1. Method

Male C57BL/6J mice aged 10–12 weeks (weight, 24–29 g) are used. We did not apply ACAS surgery to Wistar rat for several reasons. First, we don’t have appropriately sized microcoils and ameroid constrictors for rat surgery. Second, the rat model of chronic cerebral hypoperfusion has some inherent drawbacks such as optic nerve damage and difficulty in genetic assessments. Third, a poorly-developed posterior communicating artery of C57Bl/6J mouse is more suitable for maintaining chronic cerebral hypoperfusion over an extended period than Wistar rat [13,51].

**Figure 3:**
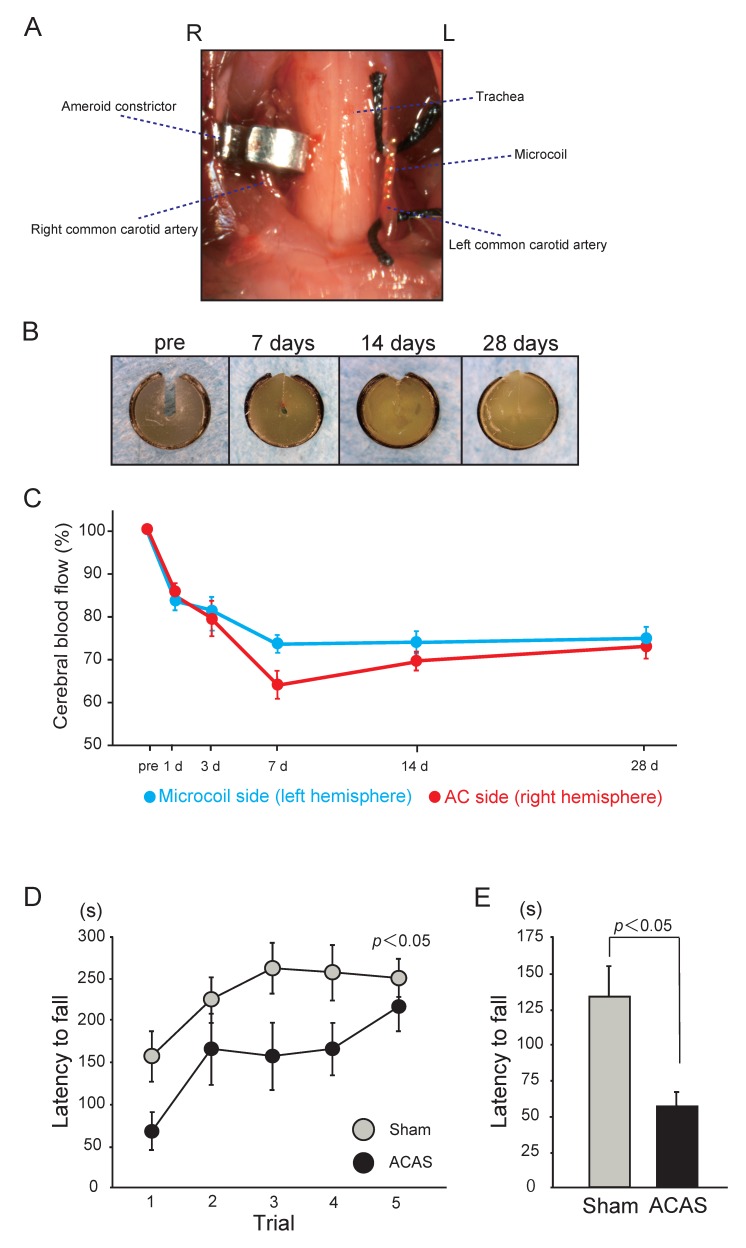
Mouse model of chronic cerebral hypoperfusion induced by asymmetric common carotid artery surgery (ACAS). (**A**) An image showing surgical implantation of the ameroid constrictor (AC) in the right common carotid artery (CCA) and the microcoil in the left CCA. (**B**) Representative images of AC at the indicated time points, showing gradual narrowing of the central lobe (lumen) with complete occlusion at 28 days. (**C**) Temporal profiles of the cerebral blood flow (CBF) in the cortex and subcortical area of surviving mice subjected to ACAS. (**D**) Behavioral performance of mice in the rotarod test after ACAS. Motor coordination and balance were tested with five consecutive trials on day 14. Data were analyzed by two-way repeated-measures analysis of variance. (**E**) Neuromuscular strength tested with the wire hang test on day 14. The error bar indicates the mean ± standard error of the mean [14].

Under anesthesia with 1.5–2% isoflurane, both CCAs are exposed and freed from their sheaths through a midline cervical incision. Two 4-0 silk sutures are placed around the distal and proximal parts of the left CCA, which is gently lifted by the sutures and placed between the loops of the microcoil (inner diameter, 0.18 mm) just below the carotid bifurcation. The microcoil is implanted by rotation around the left CCA. Then, an ameroid constrictor is surgically implanted in the right CCA (Figure 3A,B) [14]. The ameroid constrictor consists of a titanium casing surrounding a hygroscopic casein material with an internal lumen. The casein component gradually absorbs water and consequently swells, leading to predictable narrowing and occlusion of the encased arterial lumen. The inner diameter, outer diameter, and length are 0.5, 3.25, and 1.28 mm, respectively. Placement of the ameroid constrictor in the right CCA produces the expected narrowing of the inner lumen. The ameroid constrictor gradually narrows the artery for up to 28 days after the procedure, following which it results in complete occlusion (Figure 3B). The rectal temperature should be maintained between 36.5 °C and 37.5 °C during the procedure. The reported mortality rate after ACAS is approximately 20.0% [14].

The difficulty of this surgery is relatively high because it requires two techniques: ameroid constrictor placement on the right CCA and microcoil placement on the left CCA. It takes 10 min to perform this surgery. The microcoil can be purchased from Wuxi Samini Co Ltd. (Wuxi, China) and the ameroid constrictor can be purchased from Tokyo Instruments Inc. (Tokyo, Japan). The cost of one microcoil is $25 and that of an ameroid constrictor is $100. Therefore, a cost of $125 (one microcoil and one ameroid constrictor) is incurred per surgery (Table 2).

### 5.2. Cerebral Blood Flow

The reduction in CBF is generally mild, without a sharp drop in the acute phase. The acute phase observed in the BCAS model was not seen in the ACAS model, and CBF on day 1 was significantly higher than that in the BCAS model (microcoil side, 83.7%; ameroid constrictor side, 87.2%; Figure 3C) [14]. However, CBF continued to decrease after ACAS and became significantly lower than that after BCAS at 7 days (microcoil side, 74.2%; ameroid constrictor side, 64.3%). Thereafter, CBF on the microcoil side almost exhibited a plateau until day 28, whereas that on the ameroid constrictor side showed slight recovery and approached the level on the microcoil side by day 28 (microcoil side, 75.3%; ameroid constrictor side, 73.4%). However, CBF on days 14 and 28 after ACAS remained significantly lower than that after BCAS. A significant right-to-left difference was observed only on day 7 after ACAS.

### 5.3. White Matter Lesions

With regard to white matter lesions, histopathological analysis of the corpus callosum and the anterior commissure using Klüver-Barrera staining reportedly showed grade 0 or grade 1 damage at 14 days after the procedure. However, at 32 days, moderate to severe rarefaction (grade 2–3) was observed in these regions [14]. In contrast, the white matter changes in the optic tract were less severe than those in the corpus callosum and anterior commissure.

Glial activation is also observed. Relative to that after sham surgery, the demyelination in the corpus callosum after ACAS was accompanied by significant proliferation of astrocytes and microglia. Similarly, the number of astrocytes on days 14 and 32 was higher with ACAS than with sham surgery, while the microglia continued to increase in number from days 14 to 32. The number of oligodendrocytes positive for glutathione-S-transferase-pi also tended to be lower with ACAS than with sham surgery on days 14 and 32. In addition, damaged SMI32-positive axons significantly increased on day 32 relative to the number on day 14 after both ACAS and sham surgery.

With regard to white matter infarction, 88% and 75% mice in the ACAS group showed ischemic stroke at 14 and 32 days after the procedure, respectively. At 14 and 32 days, multiple infarcts developed in subcortical areas such as the corpus callosum, caudoputamen, and internal capsule on the ameroid constrictor side in 81% mice [14]. Histopathological analysis showed multiple foci of infarct-related damage in the right subcortical regions, including the corpus callosum, internal capsule, hippocampal fimbria, and caudoputamen, in 81% mice. Mice displaying such damage showed significantly poorer results in locomotor and cognitive tests.

This animal model has a limitation: ACAS mice do not develop microvasculature pathology, while in most humans, VCI results from microvasculature pathology due to hypertension, diabetes, and genetic factors such as cerebral arteriopathy with subcortical infarcts and leukoencephalopathy [14].

### 5.4. Cortical Lesions

Cortical infarcts appeared in 31% mice and accounted for 15.6% of all infarcts developed in the ACAS group at 14 and 32 days. In contrast, no infarcts were seen on the microcoil side. Hematoxylin and eosin staining showed numerous accumulations of Iba1-positive microglia and glial fibrillary acidic protein-positive astrocytes in all infarctions. Hippocampal neuronal loss was observed only on the ameroid constrictor side in 69% mice. Activated microglia surrounded the area of neuronal loss in the hippocampus [14].

### 5.5. Behavioral Analysis

Executive dysfunction was induced, and motor impairments, including gait disturbance and muscle weakness, were also observed before the development of executive dysfunction [14]. The Y-maze test showed that spatial working memory was impaired at 14 days after ACAS. Relative to that in the sham group, the rate of alternation behavior, an indicator of spatial working memory, in the ACAS group was not decreased on day 14 but significantly decreased on day 28. This indicates that ACAS specifically results in spatial working memory impairment. The number of arm entries tended to be higher in the ACAS group than in the sham group on postoperative days 14 and 28. This indicates that spontaneous activity may be increased, rather than decreased, as a result of hyperactivity in mice subjected to ACAS. Furthermore, the Morris water maze test indicated that ACAS mice showed hippocampus-dependent reference memory impairment, reflecting existing hippocampal neuronal loss.

### 5.6. Sensory/Motor Function

Importantly, the mouse ACAS model also exhibits motor impairments, including muscle weakness and gait disturbance, earlier than the BCAS model [14]. The rotarod test showed that the latency to fall was significantly shorter in the ACAS group than in the sham group on day 14; this indicates that motor coordination and balance are significantly impaired after ACAS (Figure 3D). Similarly, the latency to fall in the wire hang test was significantly shorter in the ACAS group than in the sham group on day 14, indicating significantly impaired muscle strength after ACAS (Figure 3E).

## 6. Non-Human Primate Model of Chronic Cerebral Hypoperfusion

Finally, we introduce a non-human primate model of chronic cerebral hypoperfusion. Thus far, we described rat and mouse models; however, rodent brains are different from the human brain. First, they exhibit a very small volume of white matter, which is one the reasons why most clinical trials of neuroprotective drugs have failed [50]. Although most experiments on chronic cerebral hypoperfusion are performed in rodents because of the ease of handling and greater acceptability in terms of ethics, non-human primates appear to represent the best model for the evaluation of VCI because their cerebral architecture is similar to that in humans, with a considerably larger white matter volume [70,71]. Furthermore, aging baboon brains exhibit tau pathology and amyloid β deposition [72], both of which are characteristic features of Alzheimer’s disease and cannot be found in rodent brains. Accordingly, a baboon model of chronic cerebral hypoperfusion induced by three-vessel occlusion was developed [15]. In this model, glial activation and white matter changes, which precisely mimic the pathological changes in human hypoperfusive subcortical VCI induced by chronic cerebral hypoperfusion (e.g., carotid artery stenosis [35,36], atrial fibrillation [37,38], and heart failure [39]), could be seen. Thus, this model could be useful for investigation of the pathology underlying VCI.

### 6.1. Method

**Figure 4:**
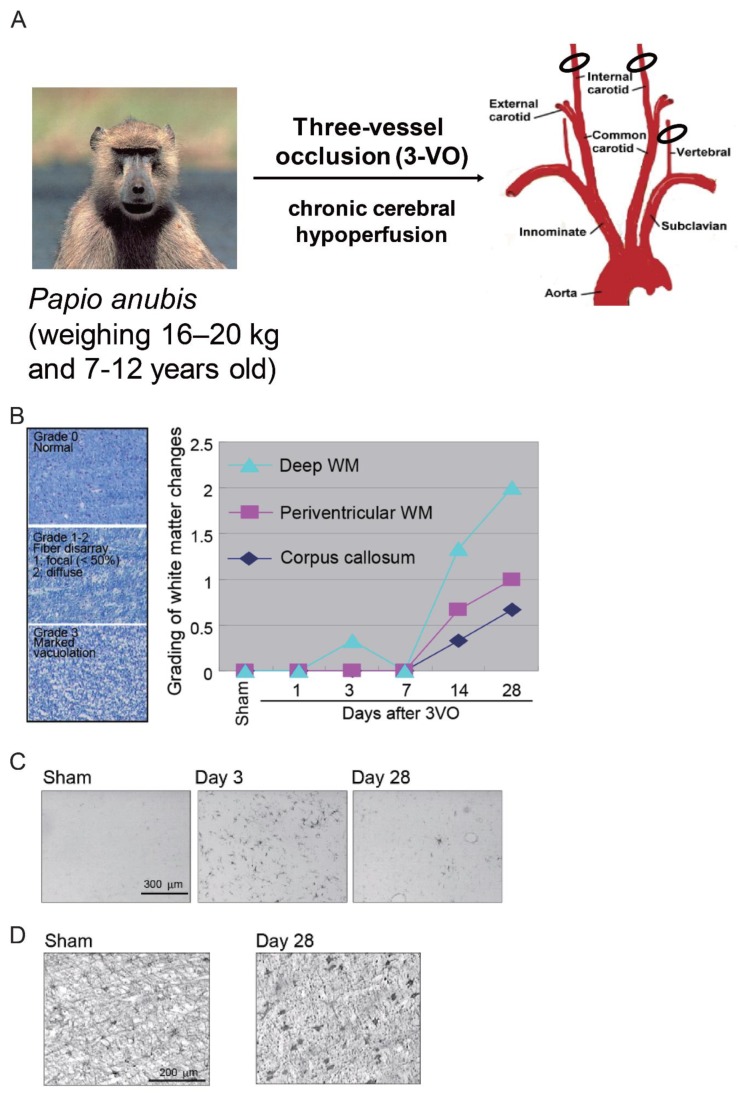
Baboon (non-human primate) model of chronic cerebral hypoperfusion induced by three-vessel occlusion (3VO). (**A**) The baboon is subjected to permanent occlusion of both the internal carotid arteries and the left vertebral artery (3VO) for 28 days. (**B**) White matter changes can be observed at 14 days after 3VO, predominantly in the deep white matter. Luxol fast blue staining shows marked vacuolation and moderate myelin loss after 14 days, with further changes continuing up to 28 days. Scale bar, 100 μm. (**C**) Immunohistochemical staining (CD68) shows microglial cell activation in the white matter in the 3VO group. Scale bar, 300 μm. (**D**) Immunohistochemical staining (glial fibrillary acidic protein) shows astroglial cell activation in the white matter in the 3VO group. Scale bar, 200 μm [15].

Adult baboons (*Papio anubis*) aged 7–12 years (weight, 16–20 kg) are used to generate this model. The baboons are subjected to permanent occlusion of both the internal carotid arteries and the left vertebral artery (three-vessel occlusion; 3VO) for 28 days (Figure 4A) [15].

This surgery is difficult: only a skilled veterinarian can perform it. The time and cost have not been well established (Table 2).

### 6.2. Cerebral Blood Flow

CBF changes in this model have not been reported and are still under investigation.

### 6.3. White Matter Lesions

White matter changes developed at 14 days after 3VO, predominantly in the deep white matter (Figure 4B). Marked vacuolation and moderate myelin loss also developed after 14 days, with changes continuing to occur up to 28 days.

Like rodent models of chronic cerebral hypoperfusion, the baboon model also showed glial activation and white matter changes after 3VO [15]. Microglial and astroglial cells were activated at three days after the procedure, with glial cell activation being more prominent in the deep white matter than in the corpus callosum and periventricular white matter (Figure 4C,D).

### 6.4. Cortical Lesions

Scattered infarcts in the cortices can be seen after 3VO. Degenerating neurons detected in the hippocampal CA1 and CA2 areas correlate with decreased neuron counts, with the greatest loss at 14 days after the procedure.

### 6.5. Behavioral Analysis

Behavioral changes are still under investigation.

### 6.6. Sensory/Motor Function

Immediately after 3VO, mild hemiparesis is temporally evident.

## 7. Conclusions

In conclusion, we described the benefits and drawbacks of four different animal models of chronic cerebral hypoperfusion. An appropriate animal model is essential for investigation of the precise mechanisms underlying VCI. The mouse model of chronic cerebral hypoperfusion induced by narrowing of the bilateral common carotid arteries with microcoils (BCAS) eventually results in hippocampal atrophy and is well validated and effective. The mouse model of chronic cerebral hypoperfusion induced by ACAS further replicates the phenotypes of human subcortical VCI, including multiple infarctions with motor and cognitive impairment. The baboon model of VCI induced by 3VO could be useful for assessing the pathophysiology of VCI because of the close resemblance to the human cerebral architecture. Finally, we expect that translational research based on the evidence generated using the mouse ACAS model or that the non-human primate model will help development of novel drugs for stroke. We also hope that these animal models will herald important breakthroughs in the development of novel treatments for VCI.

## Figures and Tables

**Table 1:** Comparison of animal models of chronic cerebral hypoperfusion.

Animal Models	Rat BCAO	Mouse BCAS	Mouse ACAS	Baboon 3-VO
CBF reduction (% preoperative level)	Rapid reduction to 30–50% of the original level with gradual recovery	Rapid reduction to 70% of the original level with gradual recovery by 0.18 mm microcoils	Gradual reduction to 60% (AC side) and 70% (microcoil side) of the original level without recovery	Under investigation
White matter lesion	Demyelination appears 14 days after surgery	Demyelination appears 14 days after surgery	Multiple infarcts and demyelination appears 14 days after surgery	Demyelination appears 14 days after surgery
Gray matter lesion	Rare	No gray matter infarction by microcoils with an inner diameter of 0.18 mm or more	Multiple infarcts only on the AC side	Rare
Cognitive dysfunction	Spatial working memory impairment	Spatial working memory impairment	Spatial working/reference memory impairment	Under investigation
Motor dysfunction	No motor deficits	No motor deficits before 3 months	Muscle weakness and gait disturbance	Temporal hemiparesis after surgery

BCAO, bilateral common carotid artery occlusion; BCAS, bilateral common carotid artery stenosis; ACAS, asymmetric common carotid artery surgery; 3-VO, three-vessel occlusion; AC, ameroid constrictor.

**Table 2:** Difficulty, time, and cost of surgery of each animal model.

Animal Models	Rat BCAO	Mouse BCAS	Mouse ACAS	Baboon 3-VO
Difficulty of surgery	Relatively easy, ligation of both CCAs	Moderate, placement of microcoils on both CCAs	Relarively high, placement of AC on the right CCA and microcoil on the left CCA	Difficult, ligation of both ICAs and left VA
Surgery time	10 min	10 min	10 min	Not established
Cost	No cost	$50 (microcoil $25 × 2)	$125 (AC $100 + microcoil $25)	Not established

BCAO, bilateral common carotid artery occlusion; BCAS, bilateral common carotid artery stenosis; ACAS, asymmetric common carotid artery surgery; 3-VO, three-vessel occlusion; CCA, common carotid artery; ICA, internal carotid artery; VA, vertebral artery; AC, ameroid constrictor.

## Abbreviations

VCI	Vascular Cognitive Impairment
CBF	Cerebral Blood Flow
CCA	Common Carotid Artery
3VO	Three-Vessel Occlusion
BCAO	Bilateral Common Carotid Artery Occlusion
BCAS	Bilateral Common Carotid Artery Stenosis
MMP	Matrix Metalloproteinase
ACAS	Asymmetric Common Carotid Artery Surgery
